# Evaluating the level of disaster preparedness of Tunisian University Hospitals using the Hospital Safety Index: a nationwide cross-sectional study

**DOI:** 10.4314/ahs.v22i3.71

**Published:** 2022-09

**Authors:** Hamdi Lamine, Naoufel Chebili, Chekib Zedini

**Affiliations:** 1 University of Sousse, Faculty of Medicine of Sousse; 2 Sahloul University Hospital; University of Sousse, Faculty of Medicine of Sousse; 3 University of Sousse, Faculty of Medicine of Sousse, Family and Community Medicine

**Keywords:** Disaster preparedness, Hospital Safety Index, Tunisian University Hospitals, Scores

## Abstract

**Background:**

Mid-way through the ‘Sendai Framework for Disaster Risk Reduction 2015–2030’, many nations are spending time, money and effort to enhance their level of preparedness facing disasters, on the other hand communities, countries and even continents are being left behind.

**Objectives:**

This study was conducted aiming at evaluating the level of disaster preparedness and response of Tunisian University Hospitals.

**Methods:**

This is a cross-sectional nationwide study conducted in Tunisia, from November 2020 to April 2021. Including 9 Tunisian University Hospitals and using the Hospital Safety Index. The data were analysed using the ‘Module and safety index calculator’.

**Results:**

This study showed that 7 out of the 9 University Hospitals were assigned the ‘B’ category of safety with overall safety indexes that ranges between 0.37 and 0.62. Also, 4 out of 9 University Hospitals had safety scores less than 0.20 regarding their emergency and disaster management.

**Conclusions:**

This is the first study to evaluate disaster preparedness and response of university hospitals in Tunisia and in north Africa. It showed that the lack of knowledge, resources and willingness, are the most important issues that needs to be addressed in order to enhance the preparedness of Tunisian hospitals.

## Introduction

Being prepared and resilient in front of disasters is an aim that started taking more attention around the globe since 1989 with the ‘International Framework for Action for the International Decade for Natural Disaster Reduction’. Since then, many local, national, and global frameworks and guidelines were created and agreed upon to help reduce the risk of disasters on humanity. One of which is the Sendai Framework for Disaster Risk Reduction 2015–2030. Currently, mid-way through this framework, decision makers, policy makers, healthcare professionals' researchers and many others are joining efforts around the globe to fulfill its ambitious goals, towards the greater aim to reduce the suffering of people. To help researchers capture the fragility of healthcare system and facilities in order to act upon them and enhance their level of preparedness, tools, such as the Hospital Safety Index, were developed and updated time after time.^1^–^7^

In the last 30 years many studies were conducted to understand and to evaluate the level of preparedness of healthcare facilities. These studies served as starting points to work on enhancing the security of hospitals thus making communities more resilient to disasters, sadly these studies, despite the fact that disaster management and resilience is a global concern, does not include all parts of the world, namely Africa in which no study was conducted to evaluate the level of preparedness of its healthcare facilities.^5^,^8^–^15^

In 2020 while Covid-19 is taking peoples' lives and generating massive influx of casualties every day around the globe, the recorded disasters were close to being the biggest number of disasters in a year, in Africa alone, floods displaced seven million residents and killed 1273 people, the largest number since 2006^16^–^18^.

Situated in north Africa, Tunisia is being, as many other countries, affected by disasters. In fact, between 2000 and 2019, 28 disasters have been reported in Tunisia, killing over 400 people and affecting nearly 1.7 million people.^18^ This is why this study was conducted aiming at evaluating the level of disaster preparedness and response of Tunisian University Hospitals.

## Methodology

### Setting

This is a nationwide cross-sectional study, conducted in Tunisia, from November 2020 to April 2021. Because of the large number of University Hospitals (UHs) in Tunisia that are not necessarily the first institutions to be in charge in case of a disaster, only UHs with a general emergency department and a bedding capacity were included in this study. A total of 9 UHs were considered.

### Survey tool

This study was conducted using the latest version (2015) of the Hospital Safety Index (HSI) containing 151 elements, each of which represents a different feature of hospital safety and is assigned one of three safety levels low (“Unlikely to function”), average (“Likely to function”), or High (“Highly likely to function”) 3.

### Data collection and analysis

Data were collected through assessments, visits and several formal interviews with the directors of the UHs, the technical directors, the Chiefs of Occupational Safety and the Chiefs of Emergency Departments (EDs). Because 'HSI’ requires the help from experts in different fields to be used properly especially for the examination of structural and non-structural elements, this examination was carried out with the assistance of each University Hospital's technical team that are in charge of all structural and non-structural components of the UHs 3.

To properly collect all data related to the 151 items of the tool, a period of approximately 20 days were spent in each UH. Photos, videos and field notes were recorded to help better analyse the findings.

The data were analysed using the ‘Module and safety index calculator’ which is an Excel file provided by WHO, which has a series of formulas that assign specific values to each element 3.

### Ethical issues

Permissions to conduct this study was obtained from the Tunisian Ministry of Health and Directors of all selected UHs. The interviewees were assured confidentiality of their data.

Also, in alliance with the WHO guidelines and recommendations, and to avoid any ethical issues the names of the UHs were coded (UH01 to UH09).

## Results

In this study 9 Tunisian UHs were included which are the biggest hospitals in Tunisia, the most multidisciplinary hospitals, and the first healthcare institutions to be involved in any national disaster. Which is in line with the WHO recommendations of first investigations of hospital preparedness level of a country. The 9 UHs are spread over 5 different Tunisian governorates representing all of the Tunisian regions (north, centre and south). Table 1, summarizes the general information and treatment capacity of the UHs.

**Table 1:** General information and treatment capacity of the hospitals

University Hospitals	Construction year	Surface	Total number of staff (2019)	Total number of beds (2019)	Average bed occupancy rate (2019) in normal situations
**UH01**	1897	140 000 m^2^	2545	1065	57.84%
**UH02**	1942	80 000 m^2^	2858	704	78.46%
**UH03**	1910	140 000 m^2^	1948	880	81,9%
**UH04**	1985	DNA*	1699	547	72.04%
**UH05**	1899	10 078 m^2^	900	180	57.2%
**UH06**	1912	120 000 m^2^	2012	995	59.09%
**UH07**	2002	DNA*	1153	414	67%
**UH08**	1991	13 000 m^2^	1647	685	72.17%
**UH09**	DNA*	DNA*	1065	494	77%

* DNA: Data Not Available

According to the data collection regarding the hazards affecting the safety of the hospitals in the included areas, floods, extreme heat and wildfires are the most common natural hazards that may occur with a high level of impact.

### Overall hospital safety

The overall safety indexes of the 9 UHs included in this study vary between 0.72 and 0.23, with only 4 UHs having a safety index greater than the medium score (0.5). Table 2 resumes the overall safety index and safety category of the 9 UHs.

**Table 2:** Safety indexes and categories of Tunisian university hospitals

University Hospitals	Safety Index	Category
**UH06**	0.72	A
**UH09**	0.62	B
**UH05**	0.59	B
**UH08**	0.51	B
**UH02**	0.47	B
**UH03**	0.4	B
**UH01**	0.37	B
**UH04**	0.37	B
**UH07**	0.23	C

### Structural safety

Many UHs are made up of older and newer buildings with inherently different safety standards, upon inspection, the vast majority of hospital buildings showed significant signs of wear, including cracks, damaged walls, damaged floors, and damaged foundations from poor and untimely repairs. Also, 4 out of the 9 UHs included are built on top of hills or high ground which expose them to strong winds, one of the UHs is built in a very low ground which expose it to floods, no specific measures to these risks were detected in either of the hospitals. Table 3 resumes the structural safety index, safety category and items contribution of the 9 UHs.

**Table 3:** Structural safety: items contributions, indexes and categories of Tunisian UHs

University Hospitals	Weighted contribution of items to the module (%)			Safety Index (bias-free)	Category

	Low	Medium	High		
**UH06**	7.5	36.5	56	0.68	a
**UH08**	4.5	43.75	51.75	0.66	a
**UH03**	30.5	29.25	40.25	0.5	b
**UH05**	34.5	26	39.5	0.48	b
**UH09**	37.5	33.75	28.75	0.4	b
**UH04**	24	56.5	19.5	0.38	b
**UH02**	43.5	49	7.5	0.24	c
**UH07**	37.5	60.25	2.25	0.22	c
**UH01**	46.75	48	5.25	0.21	c

### Non-Structural safety

Hospital entrances, windows and roofs were moderately satisfactory. Most locations of critical hospital systems and equipment are protected from potential threats. Some access routes, exits and evacuation routes are signposted and free of obstacles, unauthorized parking on the hospital grounds, natural obstacles (trees, rocks ...) or other obstacles (equipment, waste...) pose a problem, and sometimes even block emergency entrances.

External and internal communication in the UHs evaluated is provided by the national telephone network and the mobile phones of hospital staff. Alternative means of communication (erg portable radios and internal telephone networks) do not exist. All hospitals lack early warning systems to alert patients, staff and visitors of threats or disasters. None of the UHs use any hazardous liquid waste management system, wastewater as well as liquid waste from hospitals are channelled and connected with local sewerage networks. Table 4 resumes the non-structural safety index, safety category and items contribution of the 9 UHs.

**Table 4:** Non-structural safety: items contributions, indexes and categories of Tunisian UHs

University Hospitals	Weighted contribution of items to the module (%)			Safety Index (bias-free)	Category

	Low	Medium	High		
**UH06**	2.86	37.17	59.97	0.72	a
**UH08**	7.85	35.1	57.05	0.69	a
**UH09**	4.72	47.61	47.67	0.64	b
**UH04**	9.22	46.69	44.1	0.6	b
**UH05**	10.83	45.26	43.91	0.59	b
**UH03**	9.02	51.3	39.68	0.57	b
**UH02**	15.75	44.83	39.42	0.54	b
**UH01**	14.61	75.83	9.56	0.35	c
**UH07**	17.1	73.35	9.55	0.34	c

### Emergency and disaster management

Almost all hospitals did not have any disaster management plan developed or approved in accordance with the recommendations of the Ministry of Health. In most UHs, the tasks of staff in the event of a disaster are indicated. However, these tasks are not communicated to the personnel. The plans (if exists) were not tested in 8 out of the 9 UHs, no drills, exercises or simulations were made for the personnel in all of the included hospitals. No UH has either risk-specific intervention sub-plans or evacuation plans. No UH have a decontamination plan, material or procedures in place in case of biological, chemical or radiological hazards. No UH had a budget or a mechanism to obtain emergency funds. Table 5 resumes the emergency and disaster management safety index, safety category and items contribution of the 9 UHs.

**Table 5:** Emergency and disaster management: items contributions, indexes and categories of Tunisian UHs

University Hospitals	Weighted contribution of items to the module (%)			Safety Index (bias-free)	Category

	Low	Medium	High		
**UH09**	11.7	7.25	81.05	0.83	a
**UH06**	11.05	19.75	69.2	0.76	a
**UH05**	11.05	29.79	59.16	0.69	a
**UH02**	10.3	42.4	47.3	0.61	b
**UH01**	21.85	33.89	44.26	0.56	b
**UH08**	69.3	17.3	13.4	0.19	c
**UH07**	61.79	36.26	1.95	0.14	c
**UH03**	81.75	8.25	10	0.13	c
**UH04**	83.85	4.2	11.95	0.13	c

## Discussion

This study aimed at evaluating the level of disaster preparedness and response of Tunisian UHs, to the authors' knowledge, this is the first study to evaluate disaster preparedness and response of UHs in Tunisia and in North Africa.

### Overall Hospital Safety

Looking at the results 7 out of 9 UHs were classified in the second level of safety, which means for these hospitals, in the short term, intervention actions are required. The current levels of safety and emergency and disaster management at these hospitals put patients' and staff's safety, as well as the hospital's ability to function during and after emergencies and disasters, at risk^3^.

According to the literature, two possible explanations can be behind these low levels of Hospital Safety, the revolution and the socioeconomic level of Tunisia. This was the case in a similar study held in Yemen comparing the preparedness levels of hospitals before and after the Yemeni revolution among 11 hospitals has shown very weak improvement of preparedness levels between 2011 (before the revolution) and 2013 (after the revolution). Furthermore, a community's socioeconomic status affects its residents' vulnerability and medical needs in the case of a disaster. This is why, hospital disaster preparedness is influenced by socioeconomic elements such as money, legal frameworks, and health-care standards and norms^9^,^11^,^19^,^20^.

### Structural Safety

The socioeconomic status, can also explain the low level of structural safety detected in this study, in fact, disasters may completely transform the landscape of a developing country in a matter of seconds, obliterating years of progress^19^. The results of this study showed that 7 out of 9 hospitals have had a structural safety index less than 0.5, which implies that urgent intervention measures are needed regarding the structural safety of these hospitals^3^. Some hospitals included in this study could face a potential structural damage due to their location. In fact, according to the WHO the geography surrounding the hospital or health center may suggest potential hazards, such as floods in valleys or landslides on hills^21^. These risks are either unknown or neglected by the hospitals decision makers and healthcare professionals involved in disaster management, the fake feeling of security makes the structural safety of the hospital not a priority compared to other imminent issues.

The findings of this study are in line with literature explaining the low level of structural safety due to the lack of knowledge, lack of norms, and budgetary shortfall^13^,^22^–^27^, thus raising awareness about the importance of maintaining good structural safety of the facility, providing resources (mainly guidelines) and updating the knowledge of technical personnel in charge of structural components of the facility are some of the possible solutions. In addition, most of the UHs included in this study have had several expansions throughout the years, which has been proven to be one of the major causes to reduce structural safety, as it does not necessarily take into account the nature of the surrounding land and its vulnerability to natural hazards^26^. This, in Tunisia, is mainly due to lack of structured furfur planning and well performed study of needs.

### Non-structural Safety

Studies have shown that even if the hospital is structurally intact, a major damage in its non-structural components could lead to its failure in managing disasters 12,22,28,29. This study showed that only 2 out of the 9 Tunisian UHs were classified into the ‘a’ category of non-structural security. Two major issues that were detected in all of the UHs are communication systems and liquid waste management system, which are considered as critical to the smooth running of hospitals and health care institutions on a daily basis 21,30. In fact, on top of not having any alternative mean of communication, all UHs lack of means to alert patients, staff or visitors of possible threats even though some of the hospitals have the warning system in place (because of the regulations) but it has never been used or tested.

In addition to that, the fact that none of the UHs use any hazardous liquid waste management system, is posing a huge threat on the surrounding environment and the health of citizens, this needs immediate interventions knowing that hospitals too, have a social responsibility to keep the environment clean and dispose of medical waste in order to prevent pollution and illness both within and outside the hospital.

Also, because the UH's current levels of non-structural safety are such that the safety of patients and hospital staff, and the hospital's ability to function during and after emergencies and disasters, are potentially at risk,^3^ other intervention measures are needed in the short term, such as, clearing the evacuation routes from obstacles, unauthorized parking or any other possible threats to the safety of staff and patients inside the facility. Marking restricted areas and creating measures to prevent citizens, patients and visitors to access critical areas of the hospital.

### Emergency and disaster management

In this study, nearly half of the evaluated UHs (4 out of 9) were classified into the ‘c’ category of safety regarding this module with safety scores less than 0.20, which is, considered as one of the lowest scores to be recorded in the literature 9,11,14,15,31,32.

In fact, having a written disaster management plan is not enough, as this plan should be backed up by relevant hospital and health-care policies or directives that give the Hospital Disaster Committee and the designated coordinator the authority they need to plan, coordinate, and implement the hospital's disaster risk management plan. A realistic, well-thought-out disaster management strategy, one that pulls together all aspects of disaster preparedness, is the only way to ensure a rapid and effective hospital response during disasters 3,11.

Another problem cited as impacting Tunisian hospitals' disaster management was a lack of proper training for caregivers to manage such circumstances successfully in fact none of the UHs have done drills or exercises to their personnel regarding disaster management. Professionals involved in disaster management, should receive continual training, education, and drills to improve their disaster readiness and management skills^31^.

Also, according to the data collection, the Tunisian Government, does not dedicate a specific budget or mechanism to access emergency funds for hospitals, which may be the main issue if any developments in disaster preparedness plans are required. It is true that disaster preparedness and response needs a lot of financial resources but putting it the right way will help preserve more lives and more money 33,34.

On the other hand, according to the field research of this study, Tunisian decision makers and healthcare practitioners involved in disaster management pays more attention to response rather than preparedness and invest more time, money and energy in solving the problem rather than preparing for a possible scenario that is not necessarily happening, which explains the fact that none of the UHs has either risk-specific intervention sub-plans or evacuation plans.

## Limitations

Since the assessment using the HSI is based mainly on the self-judgment of the investigators, this can lead to subjectivity and perceptual bias. The authors have tried to minimize this bias by following the “WHO Guide for Evaluators” guidelines designed specifically to minimize any possible bias. Furthermore, a single evaluator has been chosen to conduct all of the evaluations, reducing the bias associated with inter-observer variability.

Also, although the study, given its national nature, is representative of all university hospitals, it does not provide a whole picture of the disaster preparedness and response of all Tunisian health system. Indeed, all levels are involved in disaster management and their preparedness level must be assessed; however, and since it is an exploratory study, the recommendations were been followed, which include focusing on UHs first to get a basic picture and a general overview of the situation.

## Conclusion

The results showed that 7 out of the 9 UHs were classified into the ‘B’ category of safety, also, nearly half of them (4 out of 9) were classified into the ‘c’ category of safety regarding their emergency and disaster management with safety scores less than 0.20, this implies that immediate assistance is required.

The lack of knowledge, resources and willingness, are the most important issues that needs to be addressed in order to enhance the preparedness of Tunisian hospitals. For that purpose, enhancing structural and non-structural safety by applying simple measures following the latest security guidelines, training caregivers routinely to update their knowledge and practice in disaster management, allocating a specific budget in each hospital for disaster management (that can cover training, preparedness actions or management), and creating an understandable, detailed national guidelines for hospital leaders to follow are some practical measures that can positively impact the level of preparedness of Tunisian hospitals facing disasters.
